# Complete Genome Sequence of Herpes Simplex Virus 1 Strain McKrae

**DOI:** 10.1128/MRA.00993-19

**Published:** 2019-09-26

**Authors:** Xiaoli Jiao, Hongyan Sui, Christopher Lyons, Bao Tran, Brad T. Sherman, Tomozumi Imamichi

**Affiliations:** a Laboratory of Human Retrovirology and Immunoinformatics, Frederick National Laboratory for Cancer Research, Frederick, Maryland, USA; b Sequencing Facility, Center for Cancer Research, Frederick National Laboratory for Cancer Research, Frederick, Maryland, USA; DOE Joint Genome Institute

## Abstract

Herpes simplex virus 1 (HSV-1) strain McKrae is highly virulent and relatively neuroinvasive in animal models compared with other wild-type HSV-1 strains. To identify the genetic determinants that lead to the unique phenotypes of the McKrae strain, we sequenced its genome with PacBio single-molecule real-time (SMRT) technology and resolved the complete sequence.

## ANNOUNCEMENT

Herpes simplex virus 1 (HSV-1) is a member of the *Alphaherpesvirinae* subfamily of the *Herpesviridae* family (1). HSV-1 strain McKrae has a double-stranded DNA genome (∼152 kb) originally isolated from a patient with herpes simplex keratitis (2). It undergoes spontaneous or induced reactivation at a higher frequency than do other HSV-1 strains (e.g., strain KOS) and is among the most virulent HSV-1 strains in mice and rabbits (3–7). Ocular infection by HSV-1 McKrae causes fatal encephalitis in approximately 50% of animals (8). The two existing genome sequences of McKrae strains (GenBank accession numbers JX142173 and JQ730035) were sequenced with Illumina and Roche 454 platforms, respectively, and ∼3.5-kb and ∼1.5-kb genomic regions were undetermined in the assemblies (8, 9).

We obtained HSV-1 strain McKrae from Jeffrey I. Cohen (National Institute of Allergy and Infectious Diseases, National Institutes of Health, Bethesda, MD). The virus was propagated in Vero cells (ATCC, Gaithersburg, MD) (10). The genomic DNA was isolated using the PureLink viral DNA minikit (Invitrogen, Carlsbad, CA), following the manufacturer’s protocol. The viral DNA shearing was carried out using an M220 sonicator (Covaris, Woburn, MA). Size selection and subsequent cleanup were performed using a combination of BluePippin (Sage Science, Beverly, MA) with a miniTUBE Red (Covaris) and AMPure PB beads (PacBio, Menlo Park, CA), respectively. After shearing, bands of ∼5 kb in length were used for library construction using the SMRTbell template prep kit 1.0 (PacBio) with two alterations, namely, an A-tailing step abd overhang adapter ligation, as opposed to blunt adapter ligation (11). The constructed libraries were then sequenced using PacBio long-read sequencing on a Sequel system using v3.0 chemistry, with a 5 pM loading concentration and a 10-hour runtime.

**FIG 1:**
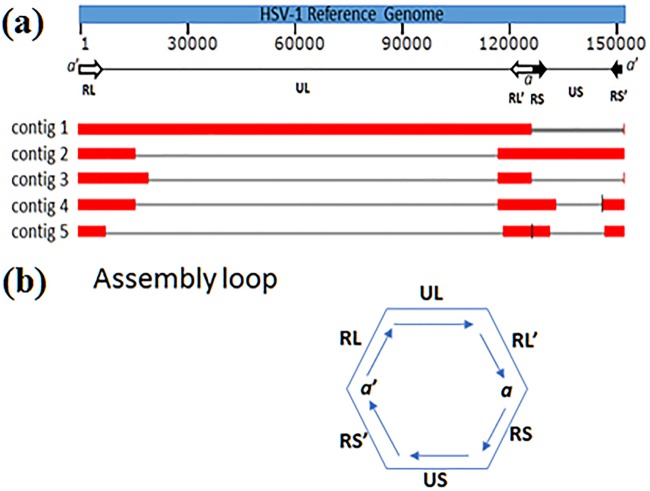
HSV-1 genome and alignments of five contigs. (a) The alignments of the five contigs against the reference genome HSV-1 strain 17. The nucleotide lengths of contigs 1, 2, 3, 4, and 5 are 126,990 bp, 44,947 bp, 20,043 bp, 33,615 bp, and 13,330 bp, respectively. The HSV-1 genome is composed of a unique long (UL) region (106.5 kb), a unique short (US) region (13.5 kb), two copies of a long inverted repeat (RL and RL’), and two copies of a short inverted repeat (RS and RS’) (6.25 kb each). The “*a*” sequence is repeated at both ends of the genome and at the internal L-S junction. There are multiple alignments for one contig due to the presence of the repeated elements in the reference genome. The gaps showing between the alignments for a contig are not deletions. (b) Assembly loop formed by the genome elements.

The HSV-1 genome (Fig. 1a) is GC rich and contains many repeat elements, including two copies of a long inverted repeat (RL) (∼8.75 kb each), two copies of a short inverted repeat (RS) (∼6.25 kb each) (8), and three copies of an “*a*” sequence, which is repeated at both ends of the genome and at the internal long-short (L-S) junction (12). Figure 1b shows a loop formed by these repeat elements which causes a complication for *de novo* assembly to resolve a full-length linear genome sequence, even with long reads. We obtained five contigs (max length, 126,990 bp; *N*_50_ length, 33,615 bp) from the PacBio Hierarchical Genome Assembly Process v4 (HGAP4) (13), with default settings, except with “seed length cutoff” set to 15 kb and “minimum required alignment length” set to 500 bp with an input genome length of 160 kb. The alignment of the five contigs (Fig. 1a) against the genome sequence of HSV-1 reference strain 17 (GenBank accession number JN555585) by BLASR (v2.0.0) (14), with default settings, shows that contig 1 (126,990 bp) and contig 2 (44,947 bp) jointly covered the full length of the reference genome from the 5′ to 3′ ends, and we merged them into a draft sequence. The genome termini were manually determined by the *a* sequence (303 bp) bracketed by two DR1 elements, which also had an inverted repeat in the L-S junction (15). The draft sequence was then refined by the PacBio genome resequencing pipeline (SMRT Analysis v6.0), with default settings (16). A total of 4,834,368 subreads (mean length, 2,183; maximum length, 26,927; total bases, 10,553,016,021) were mapped to the final resolved genome sequence. The average depth per base pair is 64,245 reads, a coverage that ensures high-quality consensus base calling (>99.99% accuracy) (13).

The finished length of the complete genome is 152,361 bases, with a G+C content of 68.21%. It resolves ∼3.7 kb of a total of 11 gaps in the two GenBank records (accession numbers JX142173 and JQ730035), including an ∼100-bp gap in the PQ repeat region of UL36. UL56 contains a single base pair insertion frameshift (amino acid 97) that results in a divergent and truncated C terminus, which was reported in JQ730035 (8) but not found in JX142173 (9). Only one coding mutation (L61P) in UL53 was found in the assembly due to a single nucleotide variation at 112,361 (T < C), but it was not detected in JX142173 and JQ730035. The high concordance (>99.8% identity) between the assembly and the two GenBank records in the nongapped regions indicates the high quality of the assembly.

### Data availability.

The complete genome sequence has been deposited at GenBank under the accession number MN136524, with SRA accession number SRR9719184. The annotations for the genes and coding sequences were transferred from HSV-1 strain 17 to the McKrae strain based on sequence homology (17, 18).
